# Neurofeedback Training for Psychiatric Disorders Associated with Criminal Offending: A Review

**DOI:** 10.3389/fpsyt.2017.00313

**Published:** 2018-01-25

**Authors:** Sandra Fielenbach, Franc C. L. Donkers, Marinus Spreen, Harmke A. Visser, Stefan Bogaerts

**Affiliations:** ^1^FPC Dr. S. van Mesdag, Groningen, Netherlands; ^2^Tilburg University, Tilburg, Netherlands; ^3^Maastricht University, Maastricht, Netherlands; ^4^FPC De Kijvelanden, Poortugaal, Netherlands

**Keywords:** neurofeedback, criminal offending, impulsivity, electroencephalographic learning, neurofeedback-learning

## Abstract

**Background:**

Effective treatment interventions for criminal offenders are necessary to reduce risk of criminal recidivism. Evidence about deviant electroencephalographic (EEG)-frequencies underlying disorders found in criminal offenders is accumulating. Yet, treatment modalities, such as neurofeedback, are rarely applied in the forensic psychiatric domain. Since offenders usually have multiple disorders, difficulties adhering to long-term treatment modalities, and are highly vulnerable for psychiatric decompensation, more information about neurofeedback training protocols, number of sessions, and expected symptom reduction is necessary before it can be successfully used in offender populations.

**Method:**

Studies were analyzed that used neurofeedback in adult criminal offenders, and in disorders these patients present with. Specifically aggression, violence, recidivism, offending, psychopathy, schizophrenia, attention-deficit hyperactivity disorder (ADHD), substance-use disorder (SUD), and cluster B personality disorders were included. Only studies that reported changes in EEG-frequencies posttreatment (increase/decrease/no change in EEG amplitude/power) were included.

**Results:**

Databases Psychinfo and Pubmed were searched in the period 1990–2017 according to the Preferred Reporting Items for Systematic Reviews and Meta-Analyses, resulting in a total of 10 studies. Studies in which neurofeedback was applied in ADHD (*N* = 3), SUD (*N* = 3), schizophrenia (*N* = 3), and psychopathy (*N* = 1) could be identified. No studies could be identified for neurofeedback applied in cluster B personality disorders, aggression, violence, or recidivism in criminal offenders. For all treatment populations and neurofeedback protocols, number of sessions varied greatly. Changes in behavioral levels ranged from no improvements to significant symptom reduction after neurofeedback training. The results are also mixed concerning posttreatment changes in targeted EEG-frequency bands. Only three studies established criteria for EEG-learning.

**Conclusion:**

Implications of the results for the applicability of neurofeedback training in criminal offender populations are discussed. More research focusing on neurofeedback and learning of cortical activity regulation is needed in populations with externalizing behaviors associated with violence and criminal behavior, as well as multiple comorbidities. At this point, it is unclear whether standard neurofeedback training protocols can be applied in offender populations, or whether QEEG-guided neurofeedback is a better choice. Given the special context in which the studies are executed, clinical trials, as well as single-case experimental designs, might be more feasible than large double-blind randomized controls.

## Introduction

### Rationale

Criminal offenders are a challenging patient group when it comes to adequate treatment interventions. This patient group exhibits externalizing behavior and usually suffers from schizophrenia, attention-deficit hyperactivity disorder (ADHD), substance-use disorder (SUD), and cluster B personality disorders, with high comorbidity rates (1, 2). In order to prevent the risk of criminal recidivism and the suffering for potential victims, effective treatment interventions are necessary.

In the last three decades, electroencephalographic (EEG)-based neurofeedback training has been increasingly used in the treatment for various psychiatric disorders. Neurofeedback is an operant conditioning training aiming to improve brain activity, as well as to improve cognitive, behavioral, and emotional self-regulatory skills by teaching patients how to control abnormal psychological states, such as inattention and stress (3, 4). Previous studies have accumulated much evidence about deviant EEG-frequencies underlying disorders commonly found in criminal offenders that could be a target for neurofeedback training. Still, to date, neurofeedback is hardly used in the forensic psychiatric domain [e.g., Ref. (5)].

In ADHD, common EEG deviations reported in the literature concern the overrepresentation of slow frequencies like delta (0.5–3.5 Hz) and theta (3.5–7.5 Hz), with reduced amplitudes of faster waves like beta (12–20 Hz) or the sensori motor rhythm (SMR, 12–15 Hz). The cortical slowing is hypothesized to underlie symptoms, such as inattention, impulsivity, and inhibitory control (6). There is an ongoing debate in the EEG-based ADHD literature about whether these deviations are more common in children presenting with ADHD rather than adults or whether there is a natural remission with aging of ADHD patients of their immature EEG activity (7). Other deviations reported include the event-related potential (ERP) markers of response preparation, specifically the contingent negative variation (CNV) component of the slow cortical potential (SCP). Aberrant CNV patterns have been related to reduce in attention, inhibition, and cognitive control (8).

While ADHD is overrepresented in forensic psychiatric patients (2), deviant EEG-frequencies have been less studied in other psychiatric disorders commonly found in criminal offenders. In schizophrenia, EEG deviations have been observed in as many as 60% of patients (9, 10). Abnormal EEG activity reported include decreased alpha activity, increased beta activity (11–13), and reduced amplitudes of the CNV, reflecting disturbed information processing (14). In SUD, chronic substance abuse has been hypothesized to produce neural changes leading to a structural state of disinhibition and impulsivity (15–17). EEG deviations found in subjects with a history of prolonged substance abuse include alterations in theta, alpha, and beta frequency bands (18, 19). These deviations in EEG-frequencies are hypothesized to underlie classic symptoms of SUD, such as craving, over-attention to drug cues, feelings of restlessness, and loss of impulse control (20–22). In antisocial personality disorder, increased slow wave activity has been observed (23); this has also been reported in borderline personality disorder (24, 25). This increase in slow wave activity has been linked to violence and aggressive behavior (26). In psychopathy, a personality construct which has many similarities with antisocial personality disorder (27), dysregulation of SCP has been linked to poor anticipatory planning, self-regulation, and formation of stable expectancies (28–31).

Although neurofeedback has been considered as a possible treatment intervention for antisocial and violent behavior [e.g., Ref. (5, 32)], not many studies have been conducted in offender populations; however, several studies indicate that improvements were found after neurofeedback training [e.g., Ref. (33–35)], as for instance, in aggressive behavior and attention (33), or even in recidivism rates (35). However, these studies did not report EEG-changes in training parameters posttreatment, so no conclusions can be drawn about how these findings are related to changes at a neurophysiological level.

Some studies suggest that greater response to neurofeedback training in terms of more successful cortical regulation will result in higher clinical improvements (6). Surprisingly, many neurofeedback studies determine the effectiveness of the training by reporting improvements in behavioral symptoms only. Whether these behavioral changes are associated with changes in cortical brain activity is not examined [e.g., Ref. (36, 37)]. Therefore, it remains unclear how many patients actually responded to the training in terms of changes in EEG activity. In addition, few studies report within-session and/or cross-session learning effects, and only focus on the pre- and post-intervention change, making it difficult to determine how many sessions were in fact necessary to reach the desired effects. Common neurofeedback protocols can range up to 50 sessions [e.g., Ref. (38, 39)], while there is also evidence suggesting that significant improvements can be achieved within as few as 15 sessions (40). The number of neurofeedback sessions required to reach optimal training success is unclear, and whether more training sessions will actually lead to higher clinical improvements is still up for debate. Reporting changes in EEG-frequency bands after neurofeedback training seems a necessary first step in determining whether treatment success was related to the applied neurofeedback protocol. Zuberer et al. (41) provide a useful review of studies that investigate learning of cortical activity in participants with ADHD and also report some studies that show non-learning, in what they call “brain-computer illiteracy” (41). Given that even studies with healthy participants have shown that about half of the participants were not able to learn cortical regulation through neurofeedback (42), it is to be expected that forensic patients with various comorbidities have more difficulties to actually learn the principles of neurofeedback. This may reduce chances to achieve beneficial clinical effects.

As forensic psychiatric patients usually present with multiple disorders (2), have difficulties adhering to long-term treatment modalities due to low levels of treatment motivation, and are highly vulnerability for psychiatric decompensation, it is important to investigate the feasibility of this intervention, before forcing a large number of sessions upon patients. More information about the type of neurofeedback training protocols, number of sessions, and expected symptom reduction is necessary.

### Research Question

This study aims to review studies that applied neurofeedback training in criminal offenders, taking into account the multiple disorders of these patients. As such, this review focuses on neurofeedback as an intervention for criminal offending, recidivism, reoffending, aggression, violence, and the following disorders associated with criminal offending: ADHD, schizophrenia, psychosis, all Cluster B personality disorders, psychopathy, and SUD. Only studies that examined whether or not neurofeedback led to changes in the trained EEG-treatment parameters were considered. Three factors contributing to the evaluation of neurofeedback training were assessed: (1) the type of neurofeedback protocol applied, (2) the number of sessions during which the neurofeedback protocol was applied, and (3) the change in neurofeedback training parameters.

## Method

### Study Design

This review focused on single-electrode EEG-neurofeedback and, therefore, excluded neurofeedback modalities, such as interhemispheric bipolar EEG-neurofeedback, near-infrared spectroscopy neurofeedback, or functional Magnetic Resonance Imaging neurofeedback. Studies in which EEG-neurofeedback was combined with other feedback modalities, such as EMG-biofeedback in the experimental condition were also excluded. Until the end of the 1990s, EEG-biofeedback was the most common search term regarding neurofeedback (43). Therefore, EEG-biofeedback was included in the search terms. The following search terms were entered into the databases: neurofeedback or EEG-neurofeedback or EEG-biofeedback AND criminal offending, recidivism, reoffending, aggression, violence, psychopathy, schizo* or psycho* or psychosis or ADHD or attention-deficit or ADD or personality disorder or antisocial or narcissistic or borderline or addict* or substance use or substance abuse or substance dependen*. Only studies using adult participants (mean age >18) were included. As the major mental disorders most commonly associated with criminal recidivism are associated with problems in impulse control and aggression, neurofeedback or EEG-neurofeedback or EEG-biofeedback AND impulsivity or aggression were included. Change in EEG-parameters was defined as whether neurofeedback resulted in a change in EEG-frequency bands (increase or decrease in mean amplitude/power). Studies in which changes in EEG training parameters were observed without highlighting the direction of the effect were excluded, as well as studies where the dependent variable was “cortical activation” or related terms without further description of specific change in trained frequency bands.

Inclusion criteria:

1. The applied treatment was EEG-neurofeedback.
2. The study contained detailed information about number of sessions applied, neurofeedback protocol applied, and electrode position used.
3. The study provided detailed information about change in EEG training parameters due to neurofeedback training.

### Search Strategy

**Figure 1:**
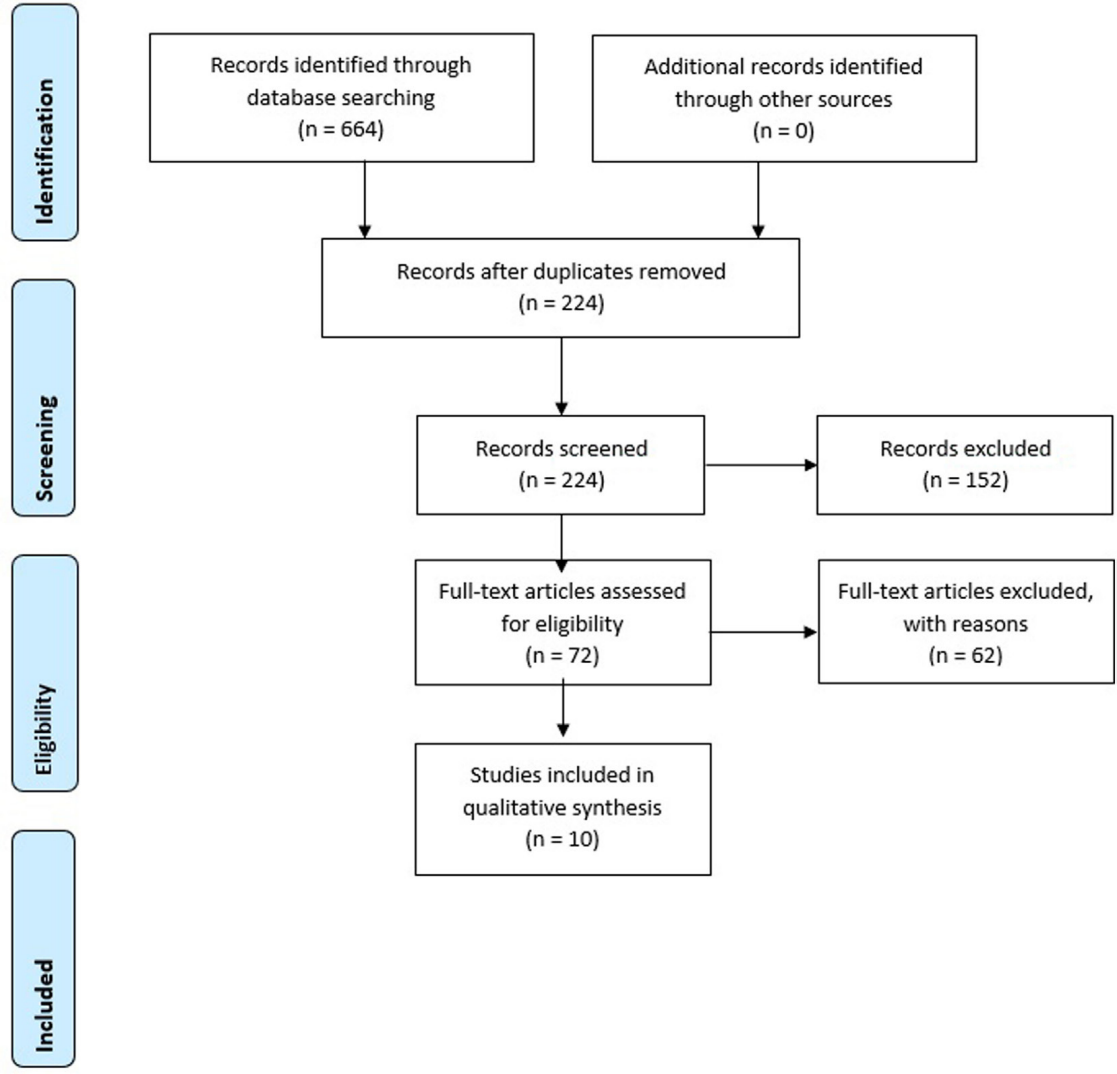
Preferred Reporting Items for Systematic Reviews and Meta-Analyses flow diagram of selection of studies. Two articles included in the search results (44, 45) refer to the same study, so the flow chart does not count them twice.

The search strategy consisted of two steps: first, databases were searched with the aforementioned terms. Electronic databases searched were PsychInfo and PubMed. Only English articles published from 1990 until November 3, 2017 were taken into account. Book chapters, dissertations, letters to the editor, and anecdotal case reports were not included. Studies in which neurofeedback protocols were tested on healthy individuals were also excluded, as well as articles describing training-effects on non-psychopathological features such as music performance. Articles resulting from the search strategy were scanned for relevance by screening titles and abstracts. Next, articles that seemed to meet inclusion criteria were examined more closely for fulfillment of all criteria. This step was done independently by two researchers (Sandra Fielenbach and Harmke A. Visser). If no agreement could be reached, an independent third party (Franc C. L. Donkers) was asked in deciding whether or not the study had to be included. See Figure 1 for a flow diagram of selection of studies.

## Results

The initial search resulted in 224 articles that were screened. Of these, 10 studies met the inclusion criteria. Table 1 lists all studies that meet the inclusion criteria and gives an overview of the employed neurofeedback protocol, characteristics of the control group, moments of measurement, targeted neuropsychological and behavioral effects, whether the study stated a criterion for defining learners and non-learners, as well as the reported results.

**Table 1:** Characteristics of the included studies (*N* = 10).

Reference, *N* (sex), medicated (yes/no)	Protocol, electrode position, number of sessions	Control group (yes/no), moment of measurement	Change in EEG-parameters investigated by	Behavioral change investigated by	Criterion established for EEG-learning (yes/no)	Results (1) Symptom change ↑ improvements (*p* < 0.05) <> = no change (2) Change in EEG-frequencies ↑ sign. increase in mean frequency ↓ sign. decrease in mean frequency <> = no change (3) Results concerning for EEG-learning
**ADHD/ADD**						

Arns et al. (46), *N* = 21, ♂/♀, yes (some patients)	QEEG-Informed protocols: beta ↑/theta ↓/alpha ↓; or beta ↓; or SMR ↑/theta ↓ (+possibly alpha ↑); or SMR ↑; individual electrode position; mean number of sessions 33.62	No, pre-training, mid-training and post-training	Changes in power in IAF, SMR, beta frequency bands and ERP measures	MINI PLUS/MINI PLUS KID, BDI (inattention, hyperactivity/impulsivity, depression scores)	No	(1) Inattention ↑, hyperactivity/impulsivity ↑, depressive symptoms ↑. Response rate was 76% (16 out of 21) on behavioral measures (2) SMR power ↓, alpha, beta <>^a^

Mayer et al. (45); Mayer et al. (44),^b^ *N* = 24, ♂/♀, yes	SCP ↓↑; Cz; 30 sessions	No, pre-, mid-, post-training and 6 months follow-up	Changes in CNV mean amplitude with Go/NoGo ERP task	ADHD-SB, WRI, FEA, FERT	Yes: learners/non-learners based on ability to differentiate between negativation/positivation in transfer condition of last 3 sessions	(1) Self-rated ADHD symptoms ↑, third-party rated ADHD symptoms ↑, depressive symptoms ↑, state and trait anxiety ↑, reaction time and reaction time variability ↑ (2) CNV showed a trend of increase over time (3) 13 learners vs 11 non-learners. Trend toward larger improvements of self-rated ADHD symptoms in learners. Higher improvements of self-rated symptoms for learners at follow-up^c^

Schönenberg et al. (40), *N* = 113, ♂/♀, yes	Theta (4–8 Hz) ↓; beta (13–21 Hz) ↑; 30 sessions	Yes: sham-NFB/meta-cognitive group therapy (MCT), pre-training, mid-training, post-training and follow-up	Changes in mean theta/beta ratio	CAARS, BDI-II, STAI-state, FPTM-23, TAP, Stroop, CPT, INKA	No	(1) Inattention ↑, hyperactivity ↑, impulsivity ↑, anxiety symptoms ↑, depression ↑, TAP flexibility ↑, reaction time <>, no superiority of NFB as compared to control groups (2) Theta/Beta ratio <>^d^

**Substance-use disorder**						

Arani et al. (18), *N* = 20, ♂, yes	Alpha (8–11 Hz) ↓/theta (5–8 Hz) ↑, after crossover alpha + theta ↑ while delta (2–5 Hz) ↓ at Pz; SMR (12–15 Hz) ↑ at Cz; 30 sessions	Yes: control group, no NFB, pre- and post-training	Changes in power of delta, theta, alpha, SMR, and high beta	SCL-90, HCQ	No	(1) SCL-90: somatization, obsession, interpersonal sensitivity, psychosis, hostility, total score ↑,^e^ HCQ: anticipation for positive outcome, desire to use, relief from withdrawal ↑, intention and plan to use <> (2) Delta ↓ (central and frontal), theta ↓ (central area), alpha ↓ (parietal and frontal areas), SMR ↑ (frontal, central area)

Horrell et al. (47), *N* = 10, ♂/♀, no	SMR (12–15 Hz) ↑ at C3/theta (4–7 Hz) ↓ at F3; 12 sessions	No, pre- and post-training	Changes in mean amplitude of theta, SMR frequency and ERP measures	BDI-II (PTSS and depressions scores), PSS-R, cue reactivity test, drug testing	No	(1) Cue reactivity test: reaction time <>, accuracy <>, depression/stress ↑, drug testing: positive drug testing ↑^a^ (2) SMR ↑ (mean increase 17%), theta <> Cue reactivity test: gamma responses to drug cues ↓

Lackner et al. (48), *N* = 25, ♂, yes	Alpha (8–12 Hz) ↑ at Pz; theta (4–7 Hz) ↑ at Fz; 12 sessions	Yes: TAU, pre- and post-training and 6 months-follow-up	Changes in absolute and relative band power for theta, alpha and beta frequency band	ACQ-R, BDI-V, BSI, FKV-lis, FPTM-23, PPR, SOC, perceived control over EEG, belief in efficacy of training	No	(1) No significant results for behavioral outcome measures posttreatment, perceived control of EEG ↑, belief in efficacy of training ↑ (2) Trend towards higher alpha, theta power ↑, beta <>^f^ No significant effects found at follow-up

**Schizophrenia**						

Gruzelier et al. (49), *N* = 25, ♂/♀, yes	SCP ↑↓; C3/C4; 10 sessions	No, improvements within and between sessions	Changes in self- regulation of interhemispheric negativity over course of training		Yes: good vs average performers based on visual inspection of performance in NFB-sessions, first 5 sessions vs last 5 sessions	(2) Ability of patients to learn self-regulation of interhemispheric negativity (3) Good performers had lateral shifts about twice as large as average performers (*p* < 0.058)^a^

Nan et al. (50), *N* = 1, ♀, yes	IAF ↑, beta 2 (20–30 Hz) ↓, 12.5 h in 4 days	No, pre and post-training	Mean relative amplitude in individual theta, alpha, sigma band, beta 1 (16–20 Hz)	Short-term memory test		(1) Memory↑ (2) Trend to increased IAB amplitude, trend toward decrease in relative beta 2 amplitude^g^

Schneider et al. (14), *N* = 24, ♂, yes (patients only)	SCP ↑↓; Cz; 20 sessions for patients, 5 for health controls	Yes: two groups, both receiving NFB: schizophrenic patients Healthy controls, pre and post-training	Changes in mean differentiation of SCP over course of training		Yes: learning success defined as mean difference between required negativity increase and negative suppression	(3) Patients were less efficient in SCP self-regulation than controls, patients were only able achieve differentiation of feedback trials comparable to controls in the last three sessions of training^a^

**Psychopathy**						

Konicar et al. (27), *N* = 14, ♂	SCP ↑↓; Fcz; 25 sessions	No, pre- and post-training	Changes in mean differentiation of SCP for first 6 sessions vs last 6 sessions	FAF, BPAQ, BIS/BAS, Flanker Test	Learning investigated, but no criteria as to group patients	(1) Physical aggression ↑, behavioral approach ↑, reaction time ↑, commission errors ↑ (2) Increase in SCP differentiation, but not for transfer conditions (3) Learning progress over the whole 25 training sessions showed a significant increase of SCP differentiation for the feedback condition as well as for the transfer condition over time^a^

*^a^No effect sizes given*.

*^b^The articles by Mayer et al. (44, 45) refer to the same study. Description is based on Mayer et al. (45)*.

*^c^Cohens’d effect size d = 1.09*.

*^d^Effect size within-participant 1:00 for NFB, 1:51 for sham, and 1:41 for mct*.

*^e^Effect sizes for significant results on the SCL-90 η^2^ ranged from 0.4 to 0.75. Effect sizes for HCQ ranged from η^2^ = 0.32 to 0.45*.

*^f^η^2^ for absolute alpha 0.139, theta 0.111*.

*^g^No effect sizes given, forward digit test improved from 7 to 9, backward digit test improved from 5 to 6*.

*N, number of participants based on initial inclusion; IAF, individual alpha frequency; MINI/MINI KID, structured ADHD interview; BDI-(II–V), German version of Beck Depression Interview; ADHD-SB, Current ADHD questionnaire as part of HASE; WRI, ADHD Wender–Reimherr Interview; FEA, ADHD symptom questionnaire; FERT, questionnaire to assess expectancy with regard to treatment; CAARS, Conners’ Adult Rating Scale; STAI, Anxiety questionnaire; FPTM-23, Therapy Motivation Questionnaire; CPT, continuous performance test; INKA, inventory for complex attention; SCL-90, Symptom Checklist-90; HCQ-45, Heroin Craving Questionnaire; PSS-R, Posttraumatic Symptom Scale—Self Report; ACQ-R, Alcohol Craving Questionnaire Revised Form; BSI, Brief Symptom Inventory; PPR, Posttraumatic Growth Inventory; SOC, Sense of Coherence Scale; FAF, Assessment of aggressiveness factors; BPAQ, Buss–Perry Aggression Questionnaire; BIS/BAS, Behavior-Inhibition/Behavior-Activation System Questionnaire; ADHD, attention-deficit hyperactivity disorder; SCP, slow cortical potential; EEG, electroencephalographic; ERP, event-related potential; CNV, contingent negative variation; SMR, sensori motor rhythm*.

Although the search concentrated on studies concerning neurofeedback training for aggression, violence, recidivism, offending, psychopathy, schizophrenia, psychosis, Cluster B personality disorders, SUD and attention-deficit disorder, only studies for schizophrenia, attention-deficit/hyperactivity disorder, and SUD could be detected that met the inclusion criteria.

### Attention Deficit/Hyperactivity Disorder

Three studies on ADHD were found that met the inclusion criteria (40, 45, 46). All studies used different neurofeedback protocols: Arns et al. (46) employed a QEEG-guided feedback protocol, where enhancement/decrease in frequencies was based on deviations found in the QEEG at pre-treatment assessment. Mayer et al. (45) employed a SCP-protocol, whereas Schönenberg et al. (40) employed a theta/beta protocol. Applied number of sessions was approximately 30. All three studies reported significant clinical changes concerning ADHD symptoms, such as inattention, hyperactivity, impulsivity, and depressive symptoms, while changes in trained EEG-frequencies posttreatment were not significant or only by trend. In Schönenberg et al. (40), no significant effect of time/treatment was found, whereas Mayer et al. (45) report a trend toward significance concerning the desired increase of CNV amplitude. In Arns et al. (46), a significantly decreased SMR power was found posttreatment in patients who underwent a SMR-training protocol, while the training was actually aimed at enhancing this frequency band. Only one of the studies actually linked the results found on a neurophysiological level to behavioral outcome measures. Arns et al. (46) reported a significant correlation between anterior individual alpha peak frequency and the percentage of improvement on depressive symptoms posttreatment, suggesting that participants with a slower anterior alpha peak frequency improved less on comorbid depressive symptoms.

Only the study by Schönenberg et al. (40) employed a control group (sham-neurofeedback and meta-cognitive therapy), and effects of neurofeedback training were not superior to effects found in the control group.

### Substance-Use Disorder

For SUD, three studies met the inclusion criteria (18, 47, 48). The studies employed three different types of protocols: a classic Peniston Protocol (alpha-theta neurofeedback) in alcohol-dependent patients (48), a Scott-Kaiser modification of the Peniston Protocol (alpha-theta training followed by a SMR-protocol) in opiate-dependent patients (18), and a SMR-based protocol in cocaine abusers (47). Number of sessions ranged from 12 to 30 sessions. In all studies, the investigated behavioral outcome measures did not only concern substance use itself but also concerned related clinical symptoms, such as broader psychopathology [e.g., the Symptom Checklist-90 (SCL-90) in the study by Arani et al. (18) and the Brief Symptom Inventory in the study by Lackner et al. (48)], posttraumatic-stress syndrome-related symptoms and depression scores [e.g., BDI in the study by Horrell et al. (47) and Lackner et al. (48)]. Posttreatment, positive effects were reported for some of the subscales of the SCL-90 (18) and depressive symptoms and level of stress (47), whereas Lackner et al. (48) found no significant behavioral changes except for an effect by trend in the sense of coherence, a concept strongly related to perceived mental health. Concerning primary symptoms of SUD, Arani et al. (18) found a significant decrease of a number of subscales of a craving questionnaire (desire to use addictive substances, relief from withdrawal symptoms and anticipation of positive outcome), and Horrell et al. (47) found a decrease in number of positive drug testing after neurofeedback training. Arani et al. (18) and Horrell et al. (47) also found significant effects in at least some of the EEG-frequency bands trained (delta, theta, alpha, and SMR). Lackner et al. (48) found a trend towards an increase in theta and alpha in absolute power bands, but the effects could not be found at 6 months follow-up assessment. However, participants’ perceived control over EEG activity, as well as anticipation of positive outcomes of training significantly, increased over the course of training.

### Schizophrenia

Three studies could be identified that met the inclusion criteria for neurofeedback studies in patients with schizophrenia (14, 49, 50). The studies by Gruzelier et al. (49) and Schneider et al. (14) employed SCP-neurofeedback at central electrode positions, whereas Nan et al. (50) trained the individual alpha peak frequency in a single-subject design. Number of sessions ranged from 10 to 20, with the exception for Nan et al. (50) who employed 12.5 h of neurofeedback training within four consecutive days. Gruzelier et al. (49) and Schneider et al. (14) investigated whether patients were able to learn to control SCP. Gruzelier et al. (49) found patients able to learn to control interhemispheric asymmetry, whereas Schneider et al. (14) found schizophrenic patients to only achieve differentiation of feedback trials comparable to controls in the last three sessions of training. Only Nan et al. (50) investigated effects on a behavioral level through a short-term memory test, which improved posttreatment, while results concerning change in EEG-frequencies posttreatment were only significant by trend.

### Offending/Psychopathy

Only one study was found regarding neurofeedback training in a population of criminal offenders and adhered to our inclusion criteria. The study by Konicar et al. (27) employed a 25-session SCP-training protocol in a population of offenders with high scores on the Psychopathy Checklist- Revised (51). Behavioral outcome measures concerned clinical symptoms, such as aggression as well as behavioral approach/avoidance constructs. Posttreatment, there was a significant reduction in physical aggression measurements as well as in behavioral approach, while reactive aggression and aggression inhibition did not improve significantly.

### EEG-Learning

Only 3 out of 10 studies established criteria for EEG-learning (14, 45, 49). Gruzelier et al. (49) differentiated between good and bad performers based on visual inspection of performance of training sessions when comparing the first five sessions with the last five sessions and reported that good performers had lateral shifts about twice as large as average performers. In Schneider et al. (14), learning success was defined as mean difference between required negativity increase and negative suppression and found that for patients, learning success took longer in time to manifest as compared to controls. Learning success correlated negatively with symptomatology at the beginning of the study, history of illness, and number of hospitalizations, implying that patients with a worse history of schizophrenic symptoms were less able to learn principals of neurofeedback training. The study by Mayer et al. (45) was the only study that established criteria for EEG-learning and also investigated whether EEG-learning was related to changes in clinical symptoms. They reported a trend toward significance for higher ADHD symptom improvement in patients who could be classified as a “neurofeedback-learner” (based on a participants’ ability to differentiate between negativation and positivation in neurofeedback transfer conditions). The study by Arns et al. (46) did not establish criteria for EEG-learning, but classified responders to neurofeedback training based on clinical symptom reduction. They found a response rate of 76% based on behavioral measures, with significant improvements on attention, impulsivity, and comorbid depressive symptoms, but posttreatment EEG measurements were only available for 6 out of 21 patients. The results of the available EEG measurements indicated changes in training parameters in an opposite direction as expected, as shown by a decrease in SMR power posttreatment when actually SMR was up-trained. In the study by Konicar et al. (27), the level of participants’ SCP differentiation was correlated with improvements on behavioral measures, indicating larger reductions in physical aggression, behavioral approach, reactive aggression, and aggression inhibition, with greater SCP differentiation indicating higher clinical improvements.

### Risk for Bias

**Figure 2:**
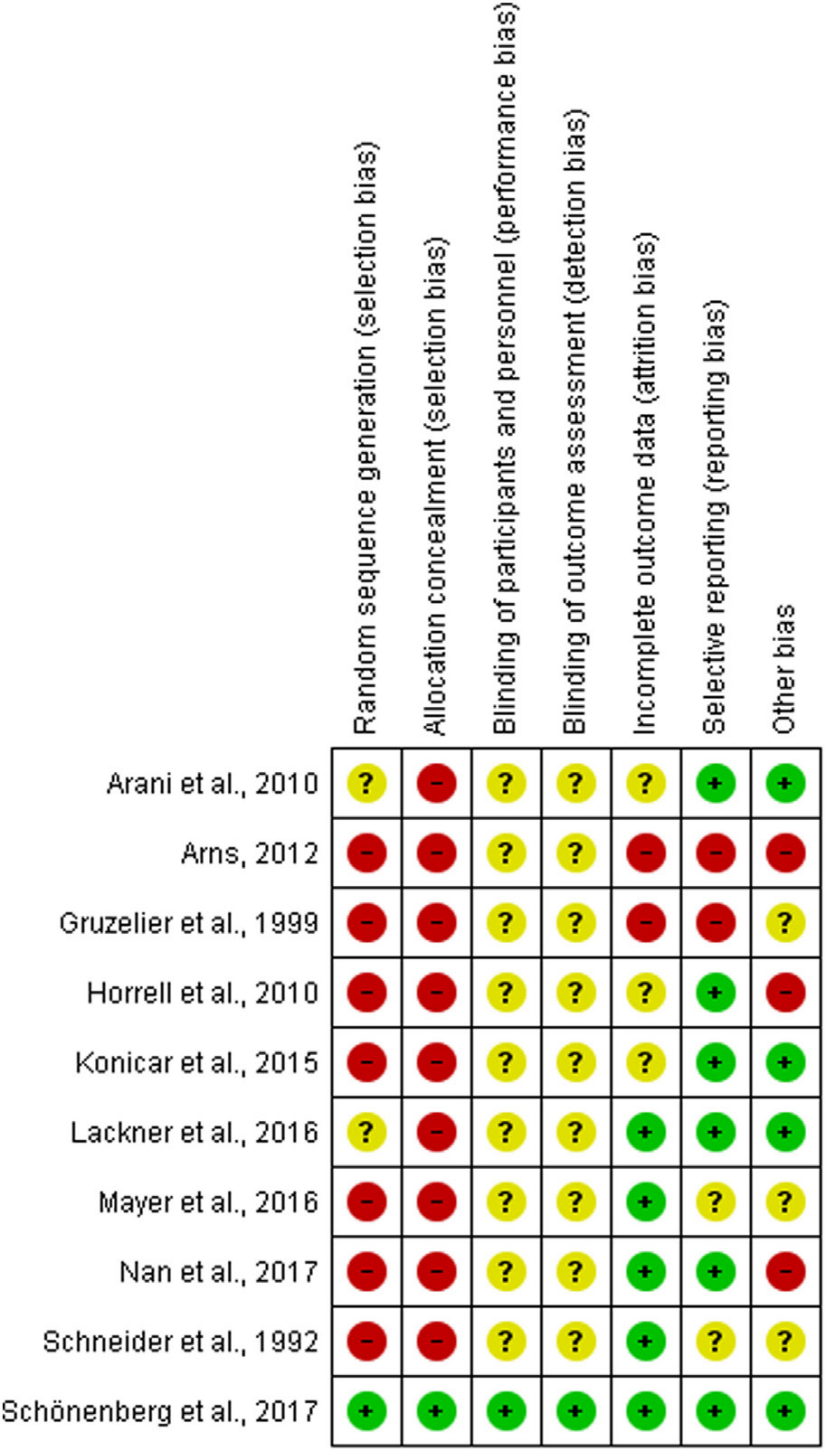
Risk for bias is assessed according to the Cochrane Handbook for Systemtic Review Intervention (52). The risk for bias is defined as ‘bias of sufficient magnitude to have a notable impact on the results or conclusions of the trial’.

**Figure 3:**
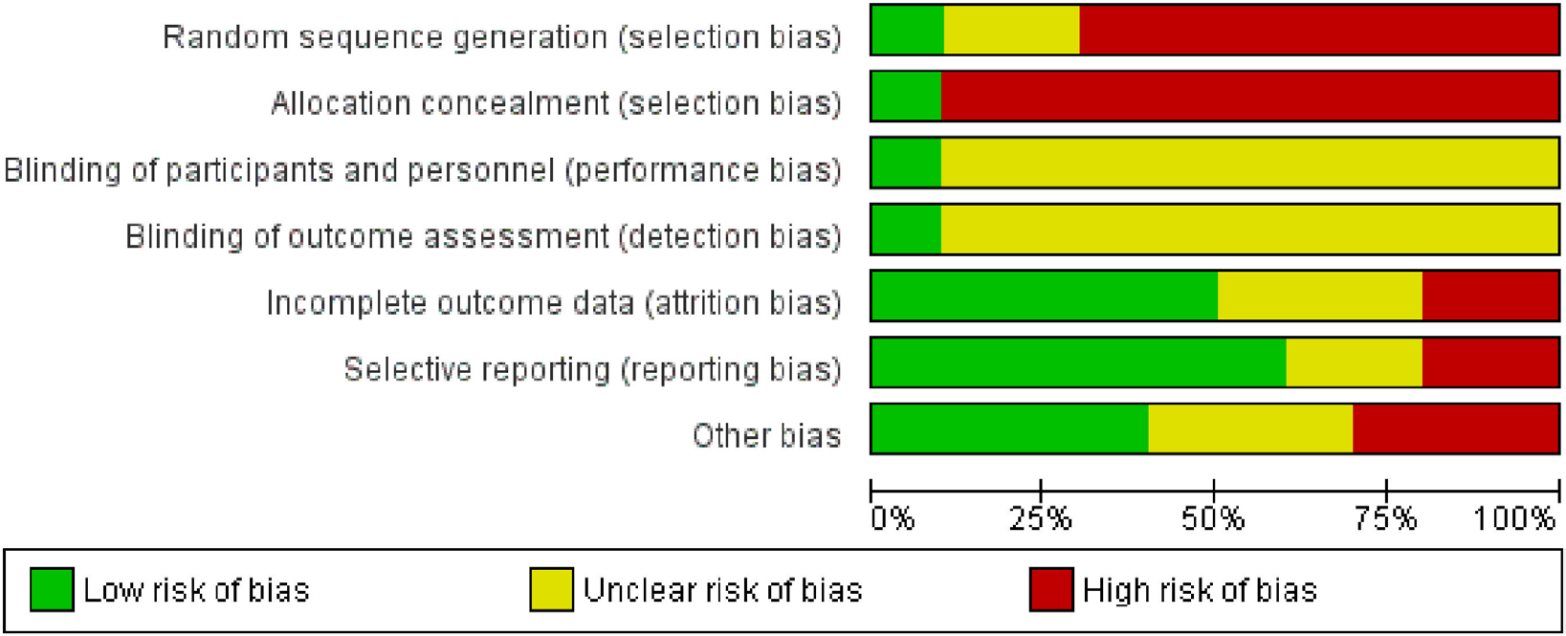
Risk of bias graph according to the Cochrane Handbook for Systemtic Review Intervention (52). Authors’ judgements about each risk of bias item presented as percentages across all included studies.

Risk for bias in the selected studies was analyzed according to Cochrane standards of practice (52). Two reviewers (Sandra Fielenbach and Harmke A. Visser) independently scored the risk for bias and then reached consensus. See Figures 2 and 3 for an assessment of bias in the included studies. Risk for bias mainly stemmed from a lack of control conditions, lack of blinding, and incomplete outcome data.

## Discussion

This study set out to review studies that applied neurofeedback in criminal offending and the disorders these patients usually present with. Only studies that described whether or not neurofeedback led to changes in trained EEG-treatment parameters were considered. To the best of our knowledge, this is the first review that investigates neurofeedback training for the purpose of applying it in the treatment of criminal offenders. The review identified 10 studies, of which three studies concentrated on neurofeedback training in patients with ADHD, three on patients with SUD, three on schizophrenia, and one on offenders with psychopathic traits. No studies fitting the inclusion criteria could be identified for neurofeedback applied in patients with cluster B personality disorders, or for reducing violence or recidivism in criminal offenders. For all treatment populations and applied neurofeedback protocols taken into account, the number of neurofeedback sessions varied greatly, ranging anywhere from 10 to 30 sessions. Most sessions were applied in patients with ADHD (about 30 sessions), whereas number of sessions was smaller in patients with schizophrenia (10–20 sessions). Possibly, patients with ADHD are more able to undergo a large number of treatment sessions then patients with schizophrenia, which are more disabled when it comes to adhering treatment due to their negative symptoms (53). In the study by Nan et al. (50), an intense 4-day neurofeedback training protocol was applied. Unfortunately, level of negative symptoms was not assessed and no indication about patient motivation for treatment was given, so it remains unclear whether individual characteristics of the patient (such as the high degree of education) contributed to the patient’s ability to follow such an intense training protocol.

With regard to the behavioral results of the studies in this review, neurofeedback research for criminal offenders might benefit most from studies where improvements were found for levels of impulsivity (40, 45, 46), psychopathy (27), hostility (18), and drug use (18, 47), which are all very often present among forensic psychiatric patients. Impulsiveness is a strong predictor of criminal offending, and the difficulties with inhibitory control make these patients more prone to aggressive outbursts and violent behavior [e.g., Ref. (54, 55)]. Substance use is associated with higher rates of violence (56). Reducing these symptoms by neurofeedback might be promising with regard to the reduction of recidivism. The results of these studies are mixed with regard to posttreatment changes in the targeted EEG-frequency bands, with results ranging from no significant changes, trends toward significance, to significant changes in the desired direction.

A central hypothesis in neurofeedback research is that the positive effects of the training are due to a feedback-driven training of specifically targeted frequency bands (40). However, even in studies where EEG-frequencies did not significantly change posttreatment (40) or even changed in the opposite direction as intended with the training protocol (46), clinical improvements could still be observed. Also, in the study by Lackner et al. (48), no behavioral improvements could be observed, while changes in theta and alpha power were significant by trend posttreatment (however, patients’ belief in the efficacy of training and the perceived control of EEG activity increased over the course of training). It is to be expected that a patients’ ability to learn principles of neurofeedback should be correlated with changes in clinical symptoms. In the study by Konicar et al. (27), level of participants’ SCP differentiation was positively correlated with improvements on behavioral measures. In Mayer et al. (45), a trend toward higher improvements of ADHD symptoms for EEG-learners could be observed. In the study by Schneider et al. (14), EEG-learning success correlated negatively with symptomatology at the beginning of the study, history of illness, and number of hospitalizations, so possibly, neurofeedback is easier for patients with less severe courses of illness. Based on the studies in this review, no final conclusion can be drawn about whether positive effects of neurofeedback are due to specific neurophysiological changes. There is still an ongoing debate about whether improvements in clinical symptoms post-neurofeedback training are due to other, non-specific effects, such as perceived self-efficacy (57), therapist–patient interaction, and/or increased ability to focus on the neurofeedback training at hand. Only four studies in this review compared the effects of neurofeedback to a control group (14, 18, 40, 48). While some of the unblinded trials in this review revealed improvements in clinical symptoms after neurofeedback training, the triple-blind, randomized controlled trial by Schönenberg et al. (40) showed no superiority of neurofeedback training over sham-neurofeedback and meta-cognitive group therapy. Most of the studies included in our review also had a high risk of bias, which was mostly due to the lack of a control group and blinding of participants and therapists. The use of adequate control groups is an ongoing debate in the literature. Sham-neurofeedback training often times contains of training of seemingly irrelevant frequency bands that are typically in the higher beta or gamma bands. However, some studies show that alterations in EEG-frequency bands posttreatment can still be observed, even though these frequency bands were not up- or down-trained during the intervention [e.g., Ref. (58)]. It is, therefore, possible that effects found in sham-neurofeedback conditions are due to training of seemingly irrelevant frequency bands. The use of an EMG-biofeedback as an adequate control group is also highly questionable, as a recent study by Barth et al. (57) showed that even EMG-biofeedback resulted in an increase of alpha power posttreatment. It is clear that more research on adequate control groups is needed.

For forensic psychiatric patients with multiple comorbidities, QEEG deviations might not match with the frequency bands that are up- or down-trained in standard neurofeedback protocols. In this review, most studies only investigated differences between groups pre-treatment, but did not investigate whether QEEG deviations at baseline actually matched the employed neurofeedback protocol. Clarke et al. (59) for example, identified three different EEG-frequency clusters in children with ADHD, who also presented with significantly different behavioral complaints between groups. They identified a subgroup who presented with increased delinquent behavior, but who showed an increased beta activity and a decreased theta activity instead of the cortical underarousal often used as an indicator for lack of inhibitory control. More research is still needed about how these EEG deviations manifest in ADHD adults, but it can be argued that these patients will most likely not profit from a standard theta/beta neurofeedback protocol.

The success of neurofeedback training for complex combinations of disorders might also be found in secondary factors such as treatment retention and teaching patients to cope with stress, rather than successfully normalizing (all) QEEG deviations. Individuals with high levels of impulsivity (such as often seen in ADHD and/or SUD) more often fail to complete treatment programs (60, 61), which in turn increases risk for recidivism. In a study by Scott et al. (39), the Scott-Kaiser modification of the Peniston Protocol was employed in subjects presenting with SUD and attention deficits, and while the study does not report outcomes on a neurophysiological level, participants remained in treatment significantly longer than controls. For criminal offenders, risk for criminal recidivism will almost certainly benefit from keeping patients in treatment. Furthermore, studies have shown that neurofeedback (especially alpha-theta protocols) can be effective in improving mentalization (62). Poor mentalization skills are believed to at least partially underlie aggressive behavior in antisocial personality disorder (63). Improving mentalization skills could serve as a protective factor toward preventing aggression among criminal offender populations (63).

None of the studies investigated in this review report serious side effects of neurofeedback training. With medication for disorder such as ADHD and schizophrenia, side effects tend to be quite stressing and uncomfortable for patients. Also, positive effects of medication tend to diminish once medication use is terminated. Often times, the efficacy of neurofeedback is questioned as it has not been shown to be superior to medication. Yet, some studies do show comparable effects of medication and neurofeedback [e.g., Ref. (37, 64)]. If similar results can be achieved with neurofeedback as with medication, neurofeedback could be seen as the less invasive treatment with less possible side effects. This would especially be the case when applied in vulnerable patient populations.

## Conclusion

More research focusing on neurofeedback and actual learning of cortical activity regulation is needed in populations with externalizing behaviors associated with violence, criminal behavior, and oftentimes multiple comorbidities. Although large randomized controlled trials are considered the gold standard in scientific research, it is questionable whether studies with criminal offenders can adhere to these strict standards, due to low levels of treatment compliance of criminal offenders making it difficult to engage these patients in scientific research (5). Clinical trials, as well as single-case experimental designs [e.g., Ref. (65)], where some compromises in research methodology and experimental controls have to be made, but where treatment is tailored to the individual and his/her clinical complaints (66) might be more feasible than large double-blind randomized controls. The study by Nan et al. (50) explored the effects of neurofeedback training in a single-subject design, but unfortunately, improvement in clinical symptoms was not investigated systematically. However, the methods used in clinical trials can provide the same level of experimental rigor and internal validity (67) if executed correctly and might help shed light on applicability of neurofeedback in criminal offenders and possibly help reduce risk of recidivism.

## Author Contributions

SF is a researcher and psychologist at FPC Dr. S. van Mesdag, situated in Groningen, the Netherlands, and a Ph.D. candidate at Tilburg University. She is the main author of this article. FD is an assistant professor at the department of cognitive neuroscience at Maastricht University, the Netherlands. MS is head of the research department of the FPC Dr. S. van Mesdag. HV is a researcher and PhD-candidate at the FPC Dr. S. van Mesdag. HV helped with screening articles for inclusion, and assessing risk of bias for the included studies. SB is a professor of Forensic Psychology at Tilburg University, the Netherlands, and head of the research department at FPC De Kijvelanden/Fivoor, located in Portugal, the Netherlands. FD, MS, and SB made important contributions to this manuscript and are supervisors to SF. FD served as a third party in deciding whether or not the study had to be included.

## Conflict of Interest Statement

The authors declare that the research was conducted in the absence of any commercial or financial relationships that could be construed as a potential conflict of interest.

## References

[1] Van Nieuwenhuizen CH Bogaerts S Ruijter EAW Bonges IL Coppens M Meijers RAAC Profiling TBS-Treatment: A Structured Cases Analysis. (TBS-behandeling geprofileerd -een gestructureerde casussenanalyse). Wetenschappelijk Onderzoek- en Documentatiecentrum (WODC). 1st ed The Netherlands: Ministry of Justice (2011).

[2] Woicik K van Lem der R Sijtsema J Bogaerts S Treatment no-shows in forensic outpatients with ADHD. Crim Behav Ment Health (2017) 27:76–88. 10.1002/cbm.1989 26887960

[3] Gunckelman JD Johnstone J Neurofeedback and the brain. J Adult Dev (2005) 12:93–100. 10.1007/s10804-005-7024-x

[4] Hammond DC Bodenhamer-Davis G Gluck G Stokes G Harper D Trudeau H Standards of practice for neurofeedback and neurotherapy: a position paper of the International Society for Neurofeedback & Research. J Neurother (2011) 15(1):54–64. 10.1080/10874208.2010.545760

[5] Van Outsem R The applicability of neurofeedback in forensic psychotherapy: a literature review. J Forens Psychiatry Psychol (2011) 22:223–42. 10.1080/14789949.2010.528012

[6] Van Doren J Heinrich H Bezold M Reuter N Kratz O Horndasch S Theta/beta neurofeedback in children with ADHD: feasibility of a short-term setting and plasticity effects. Int J Psychophysiol (2017) 112:80–8. 10.1016/j.ijpsycho.2016.11.004 27829128

[7] Mann CA Lubar JF Zimmerman AW Miller CA Muenchen RA. Quantitative analysis of EEG in boys with attention-deficit hyperactivity disorder: controlled study with clinical implications. Pediatr Neurol (1992) 8:30–6. 10.1016/0887-8994(92)90049-5 1558573

[8] Barry RJ Johnstone SJ Clarke AR. A review of electrophysiology in attention-deficit/hyperactivity disorder: II. Event-related potentials. Clin Neurophysiol (2003) 114:184–98. 10.1016/S1388-2457(02)00363-2 12559225

[9] Small JG Milstein V Sharpley PH Klapper M Small IF. Electroecephalographic findings in relation to diagnostic constructs in psychiatry. Biol Psychiatry (1984) 19:471–87. 6733170

[10] Ellingson RJ The incidence of EEG abnormality among patients with mental disorders of apparently nonorganic origin: a critical review. Am J Psychiatry (1954) 111:263–75. 10.1176/ajp.111.4.263 13197589

[11] Fenton GW Fenwick PBC Dollimore J Dunn TL Hirsch SR EEG spectral analysis in schizophrenia. Br J Psychiatry (1980) 136:445–55. 10.1192/bjp.136.5.445 7388249

[12] Merrin EL Floyd TC. Negative symptoms and EEG alpha activity in schizophrenic patients. Schizophr Res (1992) 8(1):11–20. 10.1016/0920-9964(92)90056-B 1358182

[13] Surmeli T Ertem A Eralp E Kos IA Schizophrenia and the efficacy of qEEG-guided neurofeedback treatment: a clinical case series. Clin EEG Neurosci (2012) 43(2):133–44. 10.1177/1550059411429531 22715481

[14] Schneider F Rockstroh B Heiman H Lutzenberger W Matt R Elbert T Self-regulation of slow cortical potentials in psychiatric patients: schizophrenia. Biofeedback Self Regul (1992) 17(4):277–92. 10.1007/BF01000051 1477147

[15] Bates ME Bowden SC Barry D. Neurocognitive impairment associated with alcohol use disorders: implications for treatment. Exp Clin Psychopharmacol (2002) 10:193–212. 10.1037/1064-1297.10.3.193 12233981

[16] Jentsch JD Taylor JR Impulsivity resulting from frontostriatal dysfunction in drug abuse: implication for the control of behavior by reward-related stimuli. Psychopharmacology (Berl) (1999) 146:373–90. 10.1007/PL00005483 10550488

[17] Lyvers M Loss of control in alcoholism and drug addiction: a neuroscientific interpretation. Exp Clin Psychopharmacol (2000) 8:225–49. 10.1037/1064-1297.8.2.225 10843306

[18] Arani FD Rostami R Nostratabadi M. Effectiveness of neurofeedback training as a treatment for opioid-dependent patients. Clin EEG Neurosci (2010) 41(3):170–7. 10.1177/155005941004100313 20722354

[19] Sokhadze E Stewart CM Tasman A Daniels R Trudeau D Review of rationale for neurofeedback application in adolescent substance abusers with comorbid disruptive behavioral disorders. J Neurother (2011) 15:232–61. 10.1080/10874208.2011.595298

[20] Charney DA Zikos E Gill KJ. Early recovery from alcohol dependence: factors that promote or impede abstinence. J Subst Abuse Treat (2010) 38:42–50. 10.1016/j.jsat.2009.06.002 19632079

[21] Dackis CA O’Brien CP. Cocaine dependence: a disease of the brain’s reward centers. J Subst Abuse Treat (2001) 21:111–7. 10.1016/S0740-5472(01)00192-1 11728784

[22] Volkow ND Fowler JS Wang GJ The addicted human brain: insights from imaging studies. J Clin Invest (2003) 111:1444–51. 10.1172/JCI18533 12750391 PMC155054

[23] Reyes AC Amador AA. Qualitative and quantitative EEG abnormalities in violent offenders with antisocial personality disorder. J Forensic Leg Med (2009) 16:59–63. 10.1016/j.jflm.2008.08.001 19134998

[24] De la Fuente JM Tugendhaft P Mavroudakis N. Electroencephalographic abnormalities in borderline personality disorder. Psychiatry Res (1998) 77:131–8. 10.1016/S0165-1781(97)00149-2 9541149

[25] Tanahashi Y Electroencephalographic studies of borderline personality disorder. Juntendoigaku (1988) 34:207–19.

[26] Convit A Czobor P Volavka J. Lateralized abnormality in the EEG of persistently violent psychiatric inpatients. Biol Psychiatry (1991) 30:363–70. 10.1016/0006-3223(91)90292-T 1912127

[27] Konicar L Veit R Eisenbarth H Barth B Tonin P Strehl U Brain self-regulation in criminal psychopaths. Sci Rep (2015) 5:9426. 10.1038/srep09426 25800672 PMC4371087

[28] Flor H Birbaumer N Hermann C Ziegler S Patrick CJ. Aversive pavlovian conditioning in psychopaths: peripheral and central correlates. Psychophysiology (2002) 39:505–18. 10.1017/S0048577202394046 12212643

[29] Birbaumer N Elbert T Canavan A Rockstroh B. Slow potentials of the cerebral cortex and behavior. Physiol Rev (1990) 70:1–41. 10.1152/physrev.1990.70.1.1 2404287

[30] Forth AE Hare RD. The contingent negative variation in psychopaths. Psychophysiology (1989) 26:676–82. 10.1111/j.1469-8986.1989.tb03171.x 2629015

[31] Jutai JW Hare RD. Psychopathy and selective attention during performance of a complex perceptual-motor task. Psychophysiology (1983) 20:146–51. 10.1111/j.1469-8986.1983.tb03280.x 6844513

[32] Raine A Venables PH Williams M. Relationships between N1, P300 and CNV recorded at age 15 and criminal behavior at age 24. Psychophysiology (1990) 27:567–75. 10.1111/j.1469-8986.1990.tb01978.x 2274620

[33] Martin G Johnson CL The boys Totem Town Neurofeedback Project: a pilot study of EEG biofeedback with incarcerated juvenile offenders. J Neurother (2005) 9(3):71–86. 10.1300/J184v09n03_05

[34] Smith PN Sams MW Neurofeedback with juvenile offenders: a pilot study in the use of QEEG-based and analog-based remedial neurofeedback training. J Neurother (2005) 9(3):87–99. 10.1300/J184v09n03_06

[35] Quirk DA Composite biofeedback conditioning and dangerous offenders: II. J Neurother (1995) 1(2):44–54. 10.1080/10874208.2012.10491665

[36] Duric NS Aßmus J Elgen IB. Self-reported efficacy of neurofeedback treatment in a clinical randomized controlled study of ADHD children and adolescents. Neuropsychiatr Dis Treat (2014) 10:1654–1654. 10.2147/NDT.S66466 25214789 PMC4159126

[37] Fuchs T Birbaumer N Lutzenberg W Gruzelier JH Kaiser J. Neurofeedback treatment for attention-deficit/hyperactivity disorder in children: a comparison with methylphenidate. Appl Psychophysiol Biofeedback (2003) 28(1):1–12. 10.1023/A:1022353731579 12737092

[38] Heinrich H Gevensleben H Freisleder FJ Moll GH Rothenberger A. Training of slow cortical potentials in attention-deficit/hyperactivity disorder: evidence for positive behavioral and neurophysiological effects. Biol Psychiatry (2004) 55(7):772–5. 10.1016/j.biopsych.2003.11.013 15039008

[39] Scott W Kaiser D Othmer S Sideroff I. Effects of an EEG biofeedback protocol on a mixed substance abusing population. Am J Drug Alcohol Abuse (2005) 31:455–69. 10.1081/ADA-200056807 16161729

[40] Schönenberg M Wiedemann E Schneidt A Scheeff J Logemann A Keune PM Neurofeedback, sham neurofeedback, and cognitive-behavioral group therapy in adults with attention-deficit hyperactivity disorder: a triple-blind, randomised, controlled trial. Lancet Psychiatry (2017) 4:673–84. 10.1016/S2215-0366(17)30291-2 28803030

[41] Zuberer A Brandeis D Drechsler R. Are treatment effects of neurofeedback training in children with ADHD related to the successful regulation of brain activity? A review on the learning of regulation of brain activity and a contribution to the discussion on specificity. Front Hum Neurosci (2015) 9:135. 10.3389/fnhum.2015.00135 25870550 PMC4376076

[42] Weber E Köberl A Frank S Doppelmayr M. Predicting successful learning of SMR neurofeedback in healthy participants: methodological considerations. Appl Psychophysiol Biofeedback (2011) 36:37–45. 10.1007/s10484-010-9142-x 21053066

[43] Arns M Heinrich H Strehl U. Evaluation of neurofeedback in ADHD: the long and winding road. Biol Psychol (2014) 95:108.15. 10.1016/j.biopsycho.2013.11.013 24321363

[44] Mayer K Wyckoff SN Schulz U Strehl U Neurofeedback for adult attention deficit/hyperactivity disorder: investigation of slow cortical potential neurofeedback-preliminary results. J Neurother (2012) 16(1):37–45. 10.1080/10874208.2012.650113

[45] Mayer K Blume F Wyckoff SN Schulz U Brokmeier LL Strehl U Neurofeedback of slow cortical potentials as a treatment for adults with attention-deficit/hyperactivity disorder. Clin Neurophysiol (2016) 127:1374–86. 10.1016/j.clinph.2015.11.013 26684900

[46] Arns M Drinkenburg W Kenemans JL. The effects of QEEG-informed neurofeedback in ADHD: an open-label pilot study. Appl Psychophysiol Biofeedback (2012) 37:171–80. 10.1007/s10484-012-9191-4 22446998 PMC3419351

[47] Horrell T El-Baz A Baruth J Tasman A Sokhadze G Stewart C Neurofeedback effects on evoked and induced EEG gamma band reactivity to drug-related cues in cocaine addiction. J Neurother (2010) 14(3):195–216. 10.1080/10874208.2010.501498 20976131 PMC2957125

[48] Lackner N Unterrainer H Skliris D Wood G Wallner-Lieberman SJ Neuper C The effectiveness of visual short-time neurofeedback on brain activity and clinical characteristics in alcohol use disorders: practical issues and results. Clin EEG Neurosci (2016) 47(3):188–95. 10.1177/1550059415605686 26415612

[49] Gruzelier J Hardman E Wild J Zaman R. Learned control of slow potential interhemispheric asymmetry in schizophrenia. Int J Psychophysiol (1999) 34:341–8. 10.1016/S0167-8760(99)00091-4 10610058

[50] Nan W Wan F Chang L Pun SH Vai MI Rosa A An exploratory study of intensive neurofeedback training for schizophrenia. Behav Neurol (2017) 2017:6914216 10.1155/2017/6914216 28701821 PMC5497641

[51] Hare RD Manual for the Revised Psychopathy Checklist. 2nd ed Toronto, Canada: Multi-Health Systems (2003).

[52] Higgins JPT Green S, editors. Cochrane Handbook for Systematic Reviews of Interventions Version 5.1.0. The Cochrane Collaboration (2011). Available from: http://handbook.cochrane.org

[53] Lyne J O’Donoghue B Roche E Renwick L Cannon M Clarke M Negative symptoms of psychosis: a life course approach and implications for prevention and treatment. Early Interv Psychiatry (2016) 2017:1–11. 10.1111/eip.12501 29076240

[54] Reddy LF Lee J Davis MC Altshuler L Glahn DC Miklowitz DJ Impulsivity and risk taking in bipolar disorder and schizophrenia. Neuropsychopharmacology (2014) 39(2):456–63. 10.1038/npp.2013.218 23963117 PMC3870783

[55] Swann AC Bjork JM Moeller FG Dougherty DM. Two models of impulsivity: relationship to personality traits and psychopathology. Biol Psychiatry (2002) 51(12):988–94. 10.1016/S0006-3223(01)01357-9 12062883

[56] Dugré JR Dellazizzo L Giguère C-É Potvin S Dumais A. Persistency of *Cannabis* use predicts violence following acute psychiatric discharge. Front Psychiatry (2017) 8:176. 10.3389/fpsyt.2017.00176 28983261 PMC5613094

[57] Barth B Mayer K Strehl U Fallgatter AJ Ehlis A. EMG biofeedback training in adult attention-deficit/hyperactivity disorder: an active (control) training? Behav Brain Res (2017) 329:58–66. 10.1016/j.bbr.2017.04.021 28442359

[58] Doehnert M Brandeis D Straub M Steinhausen HC Drechsler R Slow cortical potential neurofeedback in attention deficit/hyperactivity disorder: is there neurophysiological evidence for specific effects? J Neural Transm (2008) 115:1445–56. 10.1007/s00702-008-0104-x 18762860

[59] Clarke AR Barry RJ Dupuy FE Heckel LD McCarthy R Selikowitz M Behavioural differences between EEG-defined subgroups of children with attention-deficit/hyperactivity disorder. Clin Neurophysiol (2011) 122:1333–41. 10.1016/j.clinph.2010.12.038 21247797

[60] Moeller FG Dougherty DM Barratt ES Schmitz JM Swann AC Grabowski J. The impact of impulsivity on cocaine use and retention in treatment. J Subst Abuse Treat (2001) 21:193–8. 10.1016/S0740-5472(01)00202-1 11777668

[61] Wilens TE. Impact of ADHD and its treatment on substance abuse in adults. J Clin Psychiatry (2004) 65(Suppl 3):38–45. 15046534

[62] Imperatori C Della Marca G Amoroso N Maestoso G Valenti EM Massullo C Alpha/theta neurofeedback increases mentalization and default mode network connectivity in a non-clinical sample. Brain Topogr (2017) 30:822–31. 10.1007/s10548-017-0593-8 28936792

[63] Velotti P Garofalo C D’Aguanno M Petrocchi C Popolo R Salvatore G Mindfulness moderates the relationship between aggression and antisocial personality disorder traits: preliminary investigation with an offender sample. Compr Psychiatry (2016) 64:38–45. 10.1016/j.comppsych.2015.08.004 26350275

[64] Janssen TWP Bink M Weeda WD Geladé K Van Mourik R Maras A Learning curves of theta/beta neurofeedback in children with ADHD. Eur Child Adolesc Psychiatry (2016) 5:573–82. 10.1007/s00787-016-0920-8 PMC5394134 27866283

[65] Fielenbach S Donkers FCL Spreen M Bogaerts S. Neurofeedback as a treatment for impulsivity in a forensic psychiatric population with substance use disorder: study protocol of a randomized controlled trial combined with an N-of-1 clinical trial. JMIR Res Protoc (2017) 6(1):e13. 10.2196/resprot.6907 28122696 PMC5299210

[66] Rossiter TR LaVaque TJ A comparison of EEG biofeedback and psychostimulants in treating attention deficit hyperactivity disorders. J Neurother (1995) 1(1):48–59. 10.1300/J184v01n01_07

[67] Rizvi SL Nock MK. Single-case experimental designs for the evaluation of treatments for self-injurious and suicidal behaviors. Suicide Life Threat Behav (2008) 38(5):498–510. 10.1521/suli.2008.38.5.498 19014302

